# Marital Satisfaction and Depression in Mothers of 3-4 Year Old Children with Developmental Delay in Comparison with Mothers of Normal Children

**Published:** 2019

**Authors:** Mahbobeh AHMADI DOULABI, Firoozeh SAJEDI, Roshanak VAMEGHI, Mohammad Ali MAZAHERI, Alireza AKBARZADEH BAGHBAN, Fatemeh AFRAZ

**Affiliations:** 1 Midwifery and Reproductive Health Research Center, Department of Midwifery and Reproductive Health, School of Nursing and Midwifery,Shahid Beheshti University of Medical Sciences, Tehran, Iran.; 2 Pediatric Neurorehabilitation Research Center,University of Social Welfare & Rehabilitation Sciences, Tehran, Iran.; 3 Department of Education and Psychology, Faculty of Social Sciences, Shahid Beheshti University of Medical Sciences. Tehran, Iran; 4 Proteomics Research Center,Department of Basic Sciences, School of Rehabilitation Sciences,Shahid Beheshti University of Medical Sciences, Tehran, Iran

**Keywords:** Depression, Developmental delay, Marital status, Satisfaction

## Abstract

**Objectives:**

The present study evaluated the depression and marital satisfaction in mothers of 36-48 months old children with developmental delay in comparison with mothers of normal children.

**Materials & Methods:**

This cross-sectional study was performed on 616 mothers and their children, aged 36 - 48 months, from Apr 2015 to Feb 2016, in some kindergartens in Tehran, Iran. Participants were selected through multi-stage random sampling. The children were divided according to the developmental status into two groups of normal development and developmental delay. The following instruments were used: A demographic and children specification questionnaire, marital satisfaction scale, the Beck Depression Inventory, and the Ages and Stages Questionnaire. The data were analyzed using SPSS16 software. Independent *t*-test and Pearson correlation were employed at significance level of 0.05.

**Results:**

The mean age of children with developmental delay and normal development was 41.94±4.48 and 42.17±5.02 months, respectively. The prevalence of developmental delay in children aged 36-48 months was 17.4% and in normal development children was 82.6%. Developmental delay in boys was 23%. The highest incidence of developmental delays was in fine motor skills. Independent *t*-test revealed a significant difference between mothers' depression and marital satisfaction with and without developmental delays in their children (*P*=0.0001). In addition, the correlation was observed between the mother’s depression and marital satisfaction (*P*=0.0001).

**Conclusion:**

Mothers of children with developmental delay suffer more from depression and have less marital satisfaction compared to mothers of healthy children. Interventional studies to reduce depression and increase marital satisfaction and its impact on development status should be conducted.

## Introduction

Developmental disorders are one of the most common problems in children (1). From birth through age of 5 yr is the critical time in developing cognitive, social, emotional, lingual, physical and behavioral skills and it forms the basis of new skills as well as experiences in adulthood (2).

Examining the evolution of the development is done in five domains: communication, gross motor, fine motor, problem-solving, and personal-social skills (3).

A child might suffer from developmental delay in one or several domains, which may decrease their total developmental score (4). Once children fail to gain their developmental capabilities, they are diagnosed with developmental disorder (5). The risk of developmental delay depends on the relationship between biological and psychological factors (6), thus it is difficult to diagnose its etiology (7). This issue has huge impacts on their health and society at wider scale (8).

The prevalence of developmental disorders in North America and Australia is estimated between 12% and 17% and in Iran from 7%-22% (9-17), with 18% incidence among children aged 4-60 months in Tehran and in other places as 14.6% (18,19). About 8% of preschool-aged children, indicating the importance of time in diagnosis and treatment of developmental disorders (20).

A wide range of demographic causes and factors, pregnancy-related issues, or psychological, social, hereditary and environmental factors lead to developmental delay (21). It is important to recognize the risk factors (22-24).

The impact of developmental disabilities not only affects the children and their family, but it also influences society (4). Outbreak of a disease in a family member affects the whole family system and causes depression and isolation (25,26). Unfavorable life events can affect psychological well-being of an individual and lead to psychological problems such as depression and anxiety (27). 

Birth and presence of a child with mental retardation can be considered an unfavorable challenging situation for any family which consequently results in stress, frustration, sorrow, and depression (28). Parents of disabled children experience parenting stress, reduced mental health, failure in job, reduced leisure time, and higher possibility of divorce compared to parents with normal children (29-32).

The presence of a disabled child can also cause irreparable damage to mental health of the family. Parents might severely suffer from having an affected child and might experience depression, anxiety, aggression, fear and shame, or they might even wish to die (33). Parents of such children have less mental health, higher levels of anxiety, depression, and physical problems, and social performance disorder in comparison with parents of normal children (34,35). 

In view of the important role of mothers in caring, communicating with, and educating the child (36-38) and the traditional role of mothers as “caregiver”, she carries more responsibility with regard to taking care of the retarded child. Consequently, she would be exposed to more mental problems and higher risk of mental-health related problems (30, 39). 

The level of general health and psychological wellbeing of mothers of children with mental retardation is also reported lower and the anxiety higher compared to mothers with normal children (40,41). The prevalence of depression is higher among mothers of mentally retarded children (40,42). About 30%-35% of mothers with disabled children had clinical symptoms of depression and gained high scores in depression tests (43). Mothers not only have to cope with problems associated with taking care of the disabled child, but other factors cause depression which is debilitating itself, such as high costs, reduced social relationships with relatives, feeling of shame for giving birth to a disabled child, and negative attitude of others (44). 

Children’s mental retardation, disability and growth delay require special facilities for caring. All these issues pressure parents and might disturb the peace of the family. Moreover, the relationship between a disabled child and their parents might be tarnished because of child’s being separated from peers and parents’ long-term care (45). 

Furthermore, birth of a mentally retarded child disturbs psychological performances of a family. It affects mental health and targetedness of the family at macro level, and performances such as expression, conflict resolution, independence, progress, recreation, problem solving and control at micro level (46). Depression in parents with disabled children, especially mothers’, is correlated with different variables such as the type of mental retardation (47), financial resources and moderate or high socioeconomic status (48), and social support. These variables can moderate the impact of retardation (49). 

Marriage is described as the most important and fundamental human relationship as it forms the primary structure of family relationships and development of future generation (50). Marital satisfaction is a complex multidimensional phenomenon (51) widely studied by various scientific disciplines (52-59). 

An individual feels satisfied with life once their current family relationship status meets the expectations, and on the contrary, feels dissatisfied with life when their current situation in family relationships does not meet their needs (60). Several factors affect marital satisfaction including children’s health status (61, 62).

The consequences of giving birth can reduce marital relationship. Furthermore, a child can cause irritation, impatience, incompatibility, and discontent in parents (33). 

Birth and presence of a retarded child can disturb parents’ marital relationship by affecting the quality of marital relationships and changing the normal condition and interaction of family members (63). Different studies have considered presence of a child with developmental delay effective on parents’ satisfaction with life. The research results have shown significant differences between marital satisfaction of parents of retarded children and of parents of normal children (64-66). Different studies have reported the following symptoms in parents of retarded children: confusion and shock, feeling of guilt and unfulfilled wishes (64), poor psychological atmosphere, and impaired mental health of family members especially mother’s (67), higher tension (68) in parents, self-blame, having problem in adapting to and coping with child’s problems (69).

The rate of divorce is the most reliable indicator of marital disturbance (70) and it shows the difficulty in having marital satisfaction (71). In fact, divorce has a direct correlation with marital satisfaction although the rate of divorce is not a true indicator of marital satisfaction (72). Overall, 80% of divorce were reported in families with retarded children while the national statistics were indicative of less than 50% of divorce rate (73). 

Parents with disabled children experience extensive relentless stress in taking care of their children. These stressors increase the odds of marriage failure and is a risk factor for physical and psychological health of parents and children” (74). A systematic review (16 articles) was conducted to evaluate marital adjustment among parents of children with developmental delay. The results were indicative of 10 articles reporting significant differences between marital adjustment in parents with and without retarded child and six articles supporting poor marital adjustment (75).

The consequences of having a disabled child in the family such as the creation of a mentally unhealthy atmosphere (76), lower degrees of compatibility and acceptance and higher stress levels (77), psychological stress (69) and chronic depression (78) directly or indirectly decrease marital satisfaction (79). 

Health of mothers, and women in general is considered the pillar of a healthy society. Mother’s health, a key underlying factor for health of the family and society, is also the fundamental concept in socioeconomic development and wellbeing. Therefore, improving mothers’ health consequently improves community health (80). 

Considering the importance of mothers’ role in taking care, nurturing and building relationships with the child and the consequences of having developmental delay, we evaluated depression and marital satisfaction in mothers of children with and without developmental delay.

## Materials & Methods

**Study Design and Population**

This cross-sectional descriptive study was conducted from Apr 21, 2015, to Feb 20, 2016, in some kindergartens across Tehran, Iran. PASS software was employed to measure sample volume of 593 considering CI of 95% and CI length of 0.066. Since the frequency of childhood developmental delay in Iranian population has been estimated at about 20% [through literature review (*P*=0.20)] (15). Overall, 616 people were enrolled in the study, taking into account about 20% of the sample loss.

**Inclusion and exclusion criteria**

The eligible subjects were:

- Children aged 36-48 months that living with their both parents
- Without congenital malformation, developmental disorder and history of hospitalization and children.
- Mothers who had not experienced any serious stressful and unpleasant events (such as loss) for six months before the study.
- Mothers with history of children with developmental disorder” were excluded from the study. Incomplete questionnaires were completed due to excluded from the study.

**Materials & Methods**

The present cross-sectional study recruited 616 mothers and their 36-48 months old children. The children were divided according to the developmental status into two groups as normal development and developmental delay.

 The first stage of sampling employed stratified sampling technique and then every stratum was randomly selected considering the number of kindergartens in every municipal district such that 8, 17, and 10 kindergartens were selected from north, center, and south part of the city. The samples were selected purposefully from each kindergarten considering the inclusion criteria.

**Measuring tools**

The data collection instruments included parents-child demographic inventory, socioeconomic questionnaire. The demographic and obstetric inventory included parents' general information (age, educational attainment, job, gravidity and parity, and history of abortion), and Ages and Stages Questionnaire (ASQ) "the (ASQ) to determine the status of child development, Beck Depression Inventory for measuring the mothers’ depression levels, and Marital satisfaction scale (ENRICH).

The socioeconomic status was assessed by studying household monthly income; Price square feet residential ground, infrastructure, housing, number of families, parental education, number of cars, and personal computer.

The principal component analysis (PCA) was used to create SES variable. PCA is a multivariate statistical method widely used during recent years to build SES variable in studies related to health and SES. PFA (principal factor analysis) variable was an overall score and considering the qualitative nature of some variables in PCA, polychoric correlation matrix was used and an SES variable was created based on factor loadings. Then this score was categorized into five levels as follows: very low, low, moderate, high and very high (81). 

The reliability of the demographic and obstetric questionnaire was evaluated through content validity using scientific resources and experts’ opinion.

Marital satisfaction was evaluated using The Evaluation and Nurturing Relationship Issues, Communication, and Happiness scale (ENRICH). The ENRICH martial satisfaction scale, as a general measurement of marital relationships, includes idealistic distortion, marital satisfaction, personality issues, communication, conflict resolution, financial management, leisure time activities, sexual relationship, children and parenting, family and friends, equalitarian roles men, religious roles, solidarity of the couple, and marital changes. This scale has 35 items with four subscales of marital satisfaction, communication, conflict resolution, and idealistic distortion. 

The scale is scored as a five-point Likert scale. The items have the following five options with scores ranging from 1 to 5 respectively: “Strongly disagree”, “Disagree”, “Neither agree nor disagree”, “Agree”, “Strongly agree”. 

For the following questions in ENRICH couple Scales: 3, 5, 6, 7, 10, 13, 14, 18, 19, 21, 22, 23, 26, 27, 28, 29, 32, 33, 34,The scoring was reversing (1 for “Strongly Agree” and 5 for “Strongly disagree”).

This scale has four distinct scores while a total score was given to sum of the items of each scale. The raw scores were converted into percentage.

Marital satisfaction: Questions 1-5-9-13-17-21-24-30-35

Relationships: Questions 2-6-10-14-18-22-25-28-31-34

Conflict resolution: Questions 3- 7-11-15-19-23-26-29-32-33

Ideal distortion: Questions 4-8-12-16-20

The scores of each scale were calculated based on cut-off points, and the scale was interpreted based on tables of norms and interpretation guidelines (82). 

The scale alpha coefficients reported for subscales of marital satisfaction, communication, conflict resolution and idealistic distortion as 0.86, 0.80, 0.84, and 0.83, respectively. The reliability for each subscale was 0.86, 0.81, 0.90, and 0.92 in retest. Alpha coefficients of 0.86, 0.78, 0.62, and 0.77 achieved for 365 couples (82). The scale for assessing marital satisfaction used and reported its accuracy 85-95% for discriminating satisfied and dissatisfied couples (83).

For screening of depression, The Beck Depression Inventory-II (BDI-II) was used, also 21 items scoring from 0 to 63 most commonly utilized for measurement of depression.

**Beck Depression Inventory-II (BDI-II** ***)***

In order to determine depression levels the scores 0-9, 10-18, 19-30, 31-40, and 41-63 respectively indicated normal, mild, moderate, severe, and extremely severe depression. Different studies have been proved reliability of the test (84-86). In Iranian population, the internal consistency was confirmed with Cronbach's alpha of 0.87 and reliability coefficient was found at 0.74 (87). The reliability of the questionnaire was measured as 0.85, using Cronbach's alpha. 

**ASQ Questionnaire**

ASQ is currently the most widely used to determine the developmental status. Sensitivity of the ASQ test is 75% in high risk group and 100% in the community group, with specificity of 95% and 90%, respectively (88).

Validity of this test varies from 76% to 88% and includes 19 different questionnaires that can screen developmental status of children from 4 to 60 months in five different domains: communication, gross motor, fine motor, problem solving and personal-social skills. Each domain is evaluated by six questions on what the child can or cannot do. They are selected to be representatives of a developmental quotient of 75%-100%. The answer of parents to each question is “yes” to indicate that the child does the special behavior of this item, ‘‘sometimes’’ to indicate an occasional or emerging response and ‘‘not yet’’ to indicate that their child does not yet do the behavior, with a respective score of 10, 5 or 0 points. Then scores of each item summed and final score in each domain is compared to cut-off points of the ASQ guidelines. The score on any domain below the cut-off point or higher than two standard deviations below the mean of the reference group is considered abnormal and referral for further evaluation (89-93). ASQ is a reliable tool with Cronbach’s alpha of 0.86 and reliability of 0.93 for Iranian children. (94).

The reliability of this scale in present study was obtained as 0.83, using the test-retest method.

**Ethical Considerations**

Written consent forms were obtained from mothers and they were asked to fill out the following questionnaires at home in four days. This study was approved by the Ethics Committee of, University of Social Welfare & Rehabilitation Sciences (No.IR.USWR.REC.1397.100). 

**Data Analyses**

After obtaining the required permission for conducting the research, objectives were explained to authorities and instructors. The consent and cooperation of kindergarten instructors were also obtained. Demographic questionnaire of mother and child, Beck’s Depression Inventory, Enrich Marital Satisfaction Scale, and Ages and Stages Questionnaire (ASQ) according to age of the child 

The scores of ASQ were calculated with regard to cut-off points set for the age of the child. The results were reported to mothers and they were referred to special centers if the scores were lower than cut-off points. Mothers with high depression scores, according to BDI, were referred to as consultancy services. 

The data were analyzed using SPSS version 16 (Chicago, IL, USA) by t-test, Chi-square and Pearson’s correlation at significance level of 0.05.

## Results

The mean age of mothers of children with developmental delay was 30.38±5.42 yr while it was 31.63±5.50 yr in mothers with normal children. The mean age of fathers of children with developmental delay and normal children was 34.37±5.141 and 36.12±5.974 yr, respectively. Most mothers with developmental delay children had 12.25±4.22 yr of education while it was 11.88±4.714 yr for mothers of normal children. No significant difference was observed between the group of children with delayed development and normal development of their parents mean age and education (by in depended *t*-test).

The majority of mothers in both groups were housewives (63.3%) and fathers were employees (44.3%). 

Of the 616 children 36-48 months, 51.9% being female and 48.1% being male. Overall, 107 children (17.4%) were in developmental delay group and 82.6% were in normal developmental group. The median number of pregnancy was two and the number of childbirth was one in both groups so there was no significant difference between the two in these regards.

The prevalence of developmental delay was 17.4% with 12.2% and 23% in females and males, respectively. The highest prevalence of developmental delay was in fine motor (6.5%) and the lowest in personal social domains (4.2%) (Table 1) presents the rate of developmental delay in each aspect of development. 

The mean rate and SD of depression in mothers of children with developmental delay were 14.32±10.36 and it was 10.12±8.29 in mothers of normal children (*P*=0.0001). The marital satisfaction was significantly different between mothers of children with and without developmental delay with 141.41±16.25 and 147.72±12.58, respectively (*P*=0.0001). 

The results of the present study were indicative of significant differences between child gender and his/her development (*P*=0.0001), by Chi-square test and socioeconomic status (*P*=0.044) by Mann-Whitney test. The independent *t*-test showed significant differences between mother’s depression and marital satisfaction in mothers of children with and without developmental delay (Table 2). The correlation between depression and marital satisfaction was also significant (*P*=0.0001). 

**Table 1:** Frequency of developmental delay (5 domains) in children aged 36-48 months(measuring tools =ASQ)

Groups	Delay development Frequency(percent)	Normal development Frequency(percent)
Domains of development		
Communication	37 (6.0)	579 (94.0)
Gross motor	30 (4.9)	586 (95.1)
Fine motor	40 (6.5)	576 (93.5)
Problem-solving	35 (5.7)	581(94.3)
Personal-social	26 (4.2)	590 (95.8)
TOTAL	107 (17.4)	509 (82.6)

**Table 2:** Relation of mother’s depression and Marital Satisfaction developmental delay with five domains of development (measuring tools =ASQ)

Domains	Communication		Fine Motor		Gross Motor		Problem-Solving		Personal social	
	Delay (+)	Delay (-)	Delay (+)	Delay (-)	Delay (+)	Delay (-)	Delay (+)	Delay (-)	Delay (+)	Delay (-)
Depression	16.05 (10.44)	10.52 (8.67)	13.30 ( 9.2)	10.68 (8.85)	13.70 (8.42 )	10.70 (8.83)	15.60 ( 11.8)	10.57 ( 8.59)	16.66 ( 14.2)	10.61 (8.50)
Result of In depended T test	*P*=0.003		*P*=0.072		*P*=0.072		*P*=0.019		*P*=0.056	
Marital Satisfaction	140.35 (16.40)	146.08 (12.42)	142.80 (16.68)	147.94 (12.23)	141.23 (16.49)	147.00 (13.46)	141.94 (15.85)	148.98 (12.49)	140.73 (15.84)	148.63 (12/30)
Result of In depended T test	*P*=0.001		*P*=0.326		*P*=0.023		*P*=0.053		*P*=0.022	

## Discussion

The rate of developmental delay in study children was 17.4% and fine motor and personal-social domains gained the highest and lowest rates, respectively.

Overall, 18% of developmental delay was in children living in Tehran (18) while the prevalence was reported 16.2% in another study (95). The results of another study (96) are aligned with the present study with regard to domains of developmental delay (97). However, language delay was reported as the most common developmental delay (14). The reason for such differences could be the number and age of research samples (91). 

The results of the present study showed that depression in mothers of a child with developmental delay was higher than in mothers with normal children, in accordance with some studies (28, 39, 40, 44). About 56% of women had moderate and severe depression, which was indicative of high prevalence of depression among mothers with the same condition. Considering the traditional role of mothers and problems associated with taking care of disabled children, parents and especially mothers are prone to higher risk of mental health problems (30, 39). 

Sometimes the vulnerability of family to a child’s disability extremely exacerbates the mental health of the whole family. Children with depressed mothers face more cognitive, verbal and social problems compared to other children. Continuous threatening events can cause depression, and having a retarded child as such an event can decrease self-esteem and sense of self-efficiency, result in hopelessness and helplessness, and lead to different levels of depression (41). 

Furthermore, people with depression are diagnosed with lethargy and loss of interest, sense of guilt, difficulty in concentration, lack of appetite, etc. (98). Neurotransmitters such as serotonin and norepinephrine are effective in development and recurrence of depression (99). These neurotransmitters are boiamines, which play the most important role in pathophysiology of mood disorders. Serotonin is nowadays considered the most related bio amine-based neurotransmitter because of the evident impact of serotonin reuptake inhibitors in treating depression (100). Moreover, a decrease in serotonin could facilitate depression and reduce norepinephrine in postsynaptic receptors. Dopamine is also involved in pathophysiology of depression since its activity decreases as a consequence (101). 

Depression is a psychological problem that causes mood disorder and decreases libido. A correlation between outbreaks of depression symptoms and poor marital relationship is reported (102). 

The results of the present study showed that marital satisfaction in mothers of children with developmental delay was lower than that in mothers of normal children, aligned with other studies (64, 103, 104). Parents of mentally retarded children reported poor marital satisfaction (105). The odds of divorce in parents of disabled children were higher than the national statistics (73). 

On the contrary, a disabled child did not consider as a reason for marital distress (106) and another research confirmed the same (107). The reason for explaining such a difference might be the participation and cooperation of fathers in the studies.

Considering the higher responsibility of mothers in taking caring for the disabled child, the longitudinal study showed that the marital satisfaction of these mothers could increase if fathers also cooperated in caregiving (108). 

These results are confirmed by the study in which showed that sharing the responsibilities of taking care of a disabled child would be a major predictive factor in marital satisfaction. Therefore, the more the parents cooperate in taking care of their child, the more their marital satisfaction (107). 

On the other hand, 44% of parents of retarded children believed that the presence of a disabled child had strengthened their marriage and about 40% claimed that a retarded child had no effect on their marriage. It was only 21% of parents who blamed the disabled child as the cause of poor marital satisfaction (109). In Moscow, the rate of divorce in families with a disabled child was not higher than the total divorce rate (110). 

Decline in marital satisfaction is created because of the distance between status of marriage relationships and the expected favorable situation. Therefore, giving birth to a retarded child would facilitate that and lead to marital dissatisfaction of parents. On one hand, the child requires constant care and help to carry out their activities, and on the other hand, high workload of parents and modern lifestyle that created more nuclear families, might be other reasons that explain low marital satisfaction in parents of retarded children compared to parents of normal children (111). 

Marital adjustment in families could be affected by presence of a disabled child. Developing efficient communication with the spouse, absence of anger, independence of husband and wife while cooperating with each other, valuing spouse’s desires and interests, empathizing with the spouse, having similar goals and ideals, accepting difficulties and preparedness for facing life problems, staying together through happy and rough days, emphasizing on positive aspects of spouse’s personality, expressing friendship and caring for each other are important factors that guarantee continuity of marriage and marital satisfaction (33). 

Importantly, problem-solving is a skill needed by everyone since an individual would always face problems such as having a retarded child (112). The scores inefficient problem solving were significantly higher in mothers of normal children than mothers of retarded ones (63).

In this study, only the opinions of mothers have been considered. The study was conducted at Tehran's children's daycare centers; therefore, the study can be generalized to children in daycare centers in Tehran.

**In conclusion, **mothers of children with developmental delay suffer more depression and have less marital satisfaction in comparison with mothers of normal children. Therefore, interventional studies to reduce depression and increase marital satisfaction and its impact on development status should be designed. Additionally, educating parents on communication skills can improve mother’s depression and marital satisfaction. 
